# Effect of the Nano-Ca(OH)_2_ Addition on the Portland Clinker Cooking Efficiency

**DOI:** 10.3390/ma12111787

**Published:** 2019-06-02

**Authors:** Azzurra Zucchini, Paola Comodi, Alessandro Di Michele, Riccardo Vivani, Lucia Mancini, Gabriele Lanzafame, Serena Casagrande, Silvia Gentili, Francesco Vetere, Luca Bartolucci, Gianluca Polidori, Fabio Santinelli, Alessandro Neri

**Affiliations:** 1 Department of Physics and Geology, University of Perugia, Via Pascoli, 06123 Perugia, Italy; paola.comodi@unipg.it (P.C.); alessandro.dimichele@unipg.it (A.D.M.); silvia.gentili85@gmail.com (S.G.); francesco.vetere@unipg.it (F.V.); luca.bartolucci@unipg.it (L.B.); gianluca.polidori@unipg.it (G.P.); 2 Department of Pharmaceutical Sciences, University of Perugia, Via A. Fabbretti, 06123 Perugia, Italy; riccardo.vivani@unipg.it (R.V.); serena.casagrande@studenti.unipg.it (S.C.); 3 Elettra-Sincrotrone Trieste S.C.p.A., SS 14, Km 163.5 in Area Science Park, 34149 Basovizza (Trieste), Italy; lucia.mancini@elettra.eu (L.M.); gabriele.lanzafame@gmail.com (G.L.); 4 Institut of Mineralogy, Leibniz Universität Hannover, Callinstrasse 3, 30167 Hannover, Germany; 5 Colacem S.p.A., Via della Vittorina, 06024 Gubbio, Italy; f.santinelli@financo.it (F.S.); a.neri@financo.it (A.N.)

**Keywords:** Portland clinker, lime, scanning electron microscopy, X-ray diffraction, X-ray computed microtomography

## Abstract

A new technology was tested to improve the cooking efficiency of the raw mixture for Portland clinker production by the use of nano-Ca(OH)_2_. A decrease in the free lime concentration after the firing of approximately 35% and 55% in the nano-added clinkers burned at 1350 °C and 1450 °C, respectively, with respect to the standard Portland clinkers was observed. Moreover, in the nano-added clinkers, a slight decrease in alite (C3S), of approximately 2–4 wt%, and increase in belite (C2S), of approximately 5–6 wt%, were observed. Despite these variations, the C2S and C3S abundance lies within the ranges for standard Portland clinkers. The results showed that the nano-addition leads to an increase of the raw mixtures’ cooking efficiency. The relatively low energy required for the clinker firing could be used to increase the plant productivity and decrease the CO_2_ emissions during clinker burning. The decrease of the work index of the clinkers produced by the use of the nano-Ca(OH)_2_ also contributes to the energy saving during clinker grinding. Differences were also found in the pore size distribution among nano-added clinkers and the standard Portland clinker. The smallest porosities with the modal volume lying in the class of 3 × 10^−6^ mm^3^ were found to increase by the use of nano-Ca(OH)_2_. However, the pore volumes higher than 2.0 × 10^−5^ mm^3^ decreased in the nano-added clinkers.

## 1. Introduction

Portland cement is currently one of the most used construction materials with the best compromise between the high demand and the associated low cost together with its high performance and flexibility [1]. It was estimated that global cement production rose up to ~4000 Mt·yr^−1^ in 2016 [2].

The base material for cement is the clinker. It is a mixture of clays and carbonates heated up to approximately 1450 °C. During the burning process, almost all the raw materials melt, and, as a consequence, primary minerals decompose and new minerals crystallize. The four main clinker mineral phases are alite (C3S–Ca_3_SiO_5_) (In cement chemistry notation, oxides are abbreviated by the first capital letter: C = CaO, S = SiO_2_, A = Al_2_O_3_, and F = Fe_2_O_3_), belite (C2S–Ca_2_SiO_4_), celite (C3A–Ca_3_Al_2_O_6_), and ferrite [C4AF–Ca_2_(Al,Fe)_2_O_5_]. In a common Portland clinker, these minerals are all present, with the following proportions: C3S 64–45 wt %, C2S 30–10 wt %, C3A 15–5 wt %, and C4AF 12–5 wt % [3]. Minor elements also occurred in a few weight percentages within the crystal structure of the above minerals usually being, in sequence of decreasing median concentration, Mg, K, S, Na, Ti, Mn, P, Sr, F, Cl, and Cr [4].

The widespread use of the clinker as a base material for cement goes hand in hand with its huge environmental cost in terms of CO_2_ production. Two main processes contribute to the CO_2_ emission. The first one is the carbonate decomposition during the clinker burning. In fact, carbonates and, in particular, calcite, in the temperature (*T*) range from approximately 700 to 950 °C [5], at room pressure, decompose into oxides and CO_2_. The second process relies on the combustion of fuels required to achieve the high temperature needed for the clinker firing. By combining these two processes, the total CO_2_ emission from the cement industry contributes approximately to the 8% of the total anthropogenic CO_2_ production [2]. These observations, together with the widespread use of the Portland cement, claim for advanced technologies to reduce the CO_2_ emissions coming out from the Portland clinker production process. Much effort was made during past decades to increase the sustainability of the cement industry. One strategy was to reduce the amount of Portland clinker in the cement mixture by adding, on the one hand, raw materials such as meta-kaolin and/or calcite [6] and, on the other hand, by-products such as fly ashes and iron/steel slags [7,8,9,10]. Alternative materials to the Portland clinker, energetically less expensive than the standard Portland clinker, were also tested. Examples are the calcium sulfoaluminate-belite cements (CSAB) [11,12,13] which require firing *T* of approximately 1250 °C (200 °C lower than the standard Portland clinker [14]), and are produced by using by-products (e.g., fly ashes and steel slags) and synthetic gypsum in the raw mixture. Moreover, the sustainable substitution of ordinary Portland cements with the “geopolymers” has attracted the attention of many scientists during recent decades. Geopolymers are inorganic polymers that originated from the reaction of silico-aluminate either with aqueous alkali hydroxide or with silicate solution to give amorphous to semi-crystalline three-dimensional (3D) silico-aluminate structures [15,16]. The significant reduction of Greenhouse emissions during their production, together with the comparable performance [17] with respect to traditional Portland cements, make geopolymers suitable candidates to substitute ordinary Portland cements.

Among the previously mentioned approaches to “green cements”, the study of new technologies devoted to increase the sustainability of the Portland clinker production, which is well established in the European context, has rarely been taken into account. The present work was conceived on this perspective and was inspired by two main key points. The first one is the very important role of CaO in the diffusion-driven formation of the Portland clinker minerals. In fact, the Ca^2+^ self-diffusion within CaO, at first, and, later through the product layer, forms the most abundant minerals in the Portland clinker (C3S, C2S, and C3A) [18]. The second key point is connected to the size dependence of the diffusion activation energy of atoms and ions, i.e., the intrinsic diffusion coefficient increases of approximately six orders of magnitude as particles size varies from 25 to 1 nm [19]. Guided by the previously mentioned reasons, a new generation of clinkers, produced by the addition of nano-Ca(OH)_2_ to the raw mixture, was tested.

The low activation energy needed for Ca^2+^ diffusion from the nano-Ca(OH)_2_, with respect to the bigger Ca(OH)_2_ particles, is expected to enhance the clinker minerals formation during the burning process. The effects should have an impact for improving the clinker cooking efficiency and decreasing the clinker burning *T* and to enhance the sustainability of the cement production process by decreasing the CO_2_ emissions. Moreover, the partial CaCO_3_ substitution in the raw mixture by Ca(OH)_2_ will cause the decrease of the emitted CO_2_ produced by decarbonation, which further improves the clinker production sustainability. Even if nano-materials have been widely used in manufacturing industries in order to improve sustainability and performance of cement-based materials such as concrete and mortar [20,21,22,23,24,25], no studies have been performed on the nano-material addition to the clinker raw mixtures. In this paper, a new technology is designed to reduce the clinker burning *T* and partly replace carbonates by nano-Ca(OH)_2_ into the raw mixture. The chemical, mineralogical, and textural properties of the new generation of clinkers are investigated to compare with the standard Portland clinkers. A multi-analytical approach is used combining X-ray powder diffraction for the study of the mineralogical composition of the analyzed samples and Scanning Electron Microscopy coupled with the energy dispersive spectrometry that allows both the analysis of chemical compositions and the bi-dimensional (2D) image analysis of the porosity distribution. Lastly, porosity and pore size distribution are analyzed by means of X-ray computed microtomography, which is a non-destructive three-dimensional (3D) imaging technique that provides reliable information of the pore-network at the microscale [26,27].

## 2. Materials and Methods 

### 2.1. Nano-Ca(OH)_2_ Synthesis

Synthesis of nano-Ca(OH)_2_ was performed by the addition of 3 M NaOH aqueous solution to a 1.5 M CaCl_2_ aqueous solution dropwise under high power ultrasound radiation for 1 h while an inert gas (Helium) was flowing into the solution. After the synthesis, the precipitate was centrifuged and washed, by using deionized water three times to eliminate NaCl residues. An ultrasound generator used for the synthesis is a titanium horn type 750 W (20 kHz), Sonics and materials VCX750, with a tip diameter of 13 mm and with amplitude of 50%. The Ultrasound generator has a power of 750 Watt and for 1 h of synthesis produces 0.313 kg of CO_2_, according to the method IPCC GWP 100 YEARS. CaCl_2_ and NaOH were purchased from Sigma-Aldrich and used as received without further purification. The synthesized nano-Ca(OH)_2_ solution was nebulized by using the Mini Spray-dryer model B-290 (*BUCHI Italia s.r.l*, Cornaredo (MI), Italy) to obtain a dried nano-Ca(OH)_2_ powder. The spry-drying operating parameters were set as follows: inlet temperature of 135 °C, aspiration of 65%, air flow of 40 bar, and a feed rate of 10%. Ca(OH)_2_ particles were separated from the drying gas by a high-performance cyclone (Büchi, Italy).

### 2.2. Clinker Production

The raw material for Portland clinker production was marl, which occurred in the Umbria-Marche stratigraphic sequence between “scaglia variegata” and “scaglia cinerea” Formations (Italy). Marls are sedimentary rocks, with a typical grey to brownish color composed of a clay fraction originated by continental leaching and a carbonate fraction produced in shallow marine settings. The used marls were gently supplied by Colacem S.p.A. (Gubbio, Italy), which is the industrial counterpart of the present project. Colacem S.p.A. owned a marl mine located in Padule (Gubbio) where the “scaglia variegata” is extracted for the industrial clinker production. Two different marls coming from the San Marco mine (Padule) were used, called “fat marl” and “slim marl” with the former having a higher carbonate composition when compared to the latter. The two marls were mixed controlling the Lime Saturation Factor (LSF), Silica ratio (SR), and Alumina Ratio (AR) composition parameters [4] (Table 1) giving the raw mixture for the standard Portland clinker (hereafter PC), by mixing 87.0 wt % of “fat marl” and 13.0 wt % of “slim marl”, and for the Portland clinker produced by nano-Ca(OH)_2_ additions (hereafter nPC), by mixing 74.1 wt % of “fat marl”, 20.9 wt % of “slim marl”, and 5 wt % of nano-Ca(OH)_2_. The LSF, SR, and AR parameters are close for PC and nPC being variability coefficients equal to 0.03% for LSF, 0.09 for AR, and 0.15% for SR (Table 1).

The marls for the raw mixtures were ground by means of an ERWEKA AR 400 ball mill and the obtained fine powder was sieved to a maximum of 200 μm size. The raw mixtures were homogenized by using a THINKY MIXER ARE-250 (*THINKY CORPORATION*, Tokyo, Japan) and adding to the mixed powder with a 7 mm-diameter round glass marbles in order to avoid the nano-particles sticking.

The firing process was operated by means of a LINN HIGH-THERM EVA-1700 furnace (Eschenfelden, Germany) operating up to 1700 °C. Samples were prepared by mixing 25 g of raw mixture with 5.5 mL of bi-distilled water. The obtained mortar was round shaped and loaded on a refractory tile inside the furnace. Three different temperatures were chosen for clinker production (1450, 1350, and 1250 °C) in order to follow the mineral nucleation and the mineral growth during the high temperature experiments. The sample will be named, hereafter, by the raw mixture type, either PC or nPC, and the experimental temperature. nPC1450 means the nPC raw mixture is heated at 1450 °C, and so on. Our new experimental study, on the nano-Ca(OH)_2_ effect on the clinker characteristics, requires two investigation levels: (1) determine the cooking process reproducibility and (2) investigate the homogeneity of the running products. The first objective was attained by running two different samples at identical temperatures (PC1250 and PC1250_2, nPC1250 and nPC1250_2). The second objective was reached by sampling three different portions of the same experimental sample (nPC1350A, nPC1350B, and nPC1350C) for further clinker micro-analysis (see the “Clinker analysis” sub-paragraph).

The cooking process was set up starting from the procedure described by Reference [28]. The first step was the dehydration of the raw mixture, at 105 °C for 2 h. Then, *T* was raised up to 900 °C in 20 min and maintained for 1 h to allow the decarbonation reaction to take place (decarbonation step, or pre-heating [4]). The third step was the firing, which means *T* was raised up to the burning temperature (1450, 1350, or 1250 °C) in 30 min and maintained for 35 min. At the end, the refractory tile was taken out from the furnace and cooled at ambient conditions. Samples were then preserved on a drier in order to avoid the CaO re-hydration to Ca(OH)_2_.

### 2.3. Analytical Techniques

The used marls were analyzed by means of the X-ray fluorescence (XRF) technique. The analysis was carried out by an XRF spectrometer Mod. ZETIUM from Panalytical. In Table 1, the chemical composition of the marls is reported.

The synthesized nano-Ca(OH)_2_ was analyzed using Field Emission Scanning Electron Microscopy (FE-SEM), Transmission Electron Microscopy (TEM), Dynamic Light Scattering (DLS), and Brunauer–Emmett–Teller (BET) analysis.

Experimentally produced clinkers after cooling were analyzed by means of FE-SEM with laboratory X-ray computed microtomography (X-ray μCT) and X-ray Powder Diffraction (XRPD). In the following, a brief description of the used techniques and instrumentations is proposed.

#### 2.3.1. Scanning Electron Microscopy

Morphological and textural features of the formed clinker minerals as well as the synthesized nano-Ca(OH)_2_ were investigated via FE-SEM. The high-resolution backscattered electron (BSE) FE-SEM images were obtained by a Field-Emission-Gun Electron Scanning Microscope (LEO 1525) and a ZEISS AsB (Angle selective backscattered, Oberkochen, Germany) detector, available at the Department of Physics and Geology of the University of Perugia. A BrukerQuantax EDS performed the chemical analyses.

The investigation and quantification of the clinkers’ porosity was made by a bi-dimensional (2D) image analysis approach. The run-product was discriminated on BSE images (12 images and 14 images for nPC1450d and PC1350c, respectively) using the Image-ProPlus 6.0 software by applying its automatic ellipse fitting procedure. The analytical protocol for image analysis is reported in References [29,30,31,32,33]. In particular, the holes identification (in area percentage) was possible by linking the grey-level values obtained by BSE images with the sample composition. The values of magnification used in image acquisition ranged from 82× to 400× depending on the size, shape, and amount of free volume.

#### 2.3.2. Granulometric Analysis

In order to quantitatively check for the grain size of the synthesized nano-Ca(OH)_2_, TEM, and DLS analyses were performed using a Philips 208 at the Department of Physics and Geology of the University of Perugia. Moreover, DLS a Zeta Potential/Particle Sizer NICOMP™ 380 ZLS (Santa Barbara, CA, USA) instrument and BET analysis with N_2_ absorption were performed at the Department of Pharmaceutical Sciences of the University of Perugia.

#### 2.3.3. X-ray µCT Measurements and Image Processing and Analysis

The reconstruction of 3D images for samples PC1350c, nPC1350d, PC1450c, and PC1450d was obtained by laboratory X-ray μCT scanning using the TomoLab station at the Elettra synchrotron facility in Basovizza (Trieste, Italy) http://www.elettra.trieste.it/lightsources/labs-and-services/tomolab/tomolab.html) [34,35]. With regard to the clinkers burned at 1250 °C, they are very sensitive to the reaction of CaO with atmosphere (dusting). Even if they were stored in drying conditions, they completely lose the compactness at the time of X-ray μCT analysis and, thus, it was not possible to apply this technique.

The used CT system is equipped with a sealed microfocus X-ray source (L9181, Hamamatsu, Japan) and delivers a polychromatic beam in a Voltage range of 40 to 130 kV with a maximum current of 300 μA and a minimum focal spot size of 5 μm. The used detector consisted of a full frame CCD imager (4008 × 2672 pixels) coupled to a Gadox scintillator by a fiber-optic taper. The water-cooled CCD camera has a 12-bit dynamic range and an effective pixel size of 12.5 × 12.5 μm^2^. Due to the cone-beam geometry, it is possible to achieve a spatial resolution close to the focal spot size, on samples from a few millimeters to a few centimeters in size. The experimental parameters used for the X-ray μCT scans are reported in Table S1 (Supplementary materials). The slice reconstruction was performed using the commercial software COBRA (Exxim, Pleasanton, CA, USA) based on the Feldkamp algorithm [36], which also allows us to correct beam hardening artefacts [37]. The ring artefacts removal was performed by using the Pore3D software library developed at Elettra [38].

Image processing and analysis were performed on suitable Volumes of Interest (VOIs) extracted from the original imaged volumes. VOIs were filtered by an anisotropic diffusion filter in order to remove noise and facilitate the pore edges detection [39] and are then segmented by the 3D automatic MultiOtsu method [40], implemented in Pore3D, and manually adjusting the threshold values after visual inspection. By this step, binary volumes containing only the porous phase were extracted for quantitative analysis. Their representativeness was tested to determine the Representative Elementary Volumes (REVs), as proposed by Costanza-Robinson et al. [41] and applying the box-counting method [42]. For each sample, the parameters of Pore3D Basic Analysis module [38] were considered for REV determination (see recent application on porous materials [43,44]). The quantitative analysis was carried out using the Pore3D software that allowed the (i) removal of noise and background objects <3 voxels by a cycle of erosion/dilation, (ii) the calculation of the density, specific surface area, integral of mean curvature, and Euler characteristic of the pore phase [38], and (iii) the measurement of the volume of each pore (see Reference [38] for a detailed description). The 3D visualization, through volume rendering, of the analyzed samples were obtained by the commercial software VGStudio MAX 2.0 (Volume Graphics).

#### 2.3.4. XRPD Analyses

The XRPD analyses were performed by means of the Philips X’PERT MPD with X’CELERATOR detector. The Cu Kα radiation (λ = 1.54184 Å) was used together with a step scan and step time of 0.02°/step and 100 s/step, respectively. Each sample was loaded onto a zero-background quartz mono-crystal sample-holder. The Rietveld refinement method [45,46,47,48] was used (i) to perform the quantitative mineralogical analysis of the clinkers and (ii) to study the micro-structure characteristics of the synthesized nano-Ca(OH)_2_. The clinkers quantitative analysis was performed by means of the TOPAS Academic software [49,50]. The refined parameters during Rietveld refinement were: background (fitted with a 25-terms Chebyshev polynomial function), sample absorption, average crystallite size, preferred orientation, scale factor, and unit cell parameters. In order to check for the amorphous content in the analyzed samples, the external standard method was used by the addition of 10 wt % of Si to the powders obtained from the samples. The GSAS-EXPGUI (General Structure Analysis System) software [51] was employed for the microstructure analysis of the nano-Ca(OH)_2_ synthesis products by using the Lanthanum hexaboride (LaB_6_) as standard reference material. The refined parameters were background (fitted with a 16-terms Chebyshev polynomial function), sample absorption, profile function (by using a pseudo-Voigt function), scale factor, and unit cell parameters.

## 3. Results

### 3.1. Nano-Ca(OH)_2_

**Figure 1:**
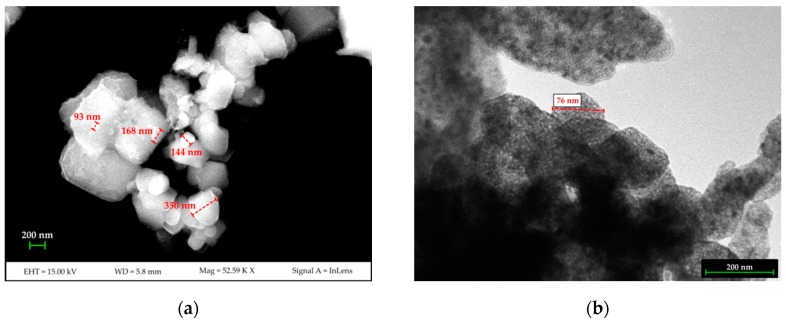
FESEM (**a**) and TEM (**b**) images of the synthesized nano-Ca(OH)_2_.

Results from the DLS analysis show that two populations are present in the synthesized nano-Ca(OH)_2_: the first is approximately 100 nm in diameter with intensity weighted relative distribution of ~18%. The second population, with an intensity weighted relative distribution of ~82%, is approximately 500 nm in size. The FE-SEM and TEM measurements (Figure 1) confirmed the nano-structure of the material and showed the two grain-populations being the smallest one as isolated nano-sized grains, and the largest one as sub-micron agglomerates made up of nano-grains.

**Figure 2:**
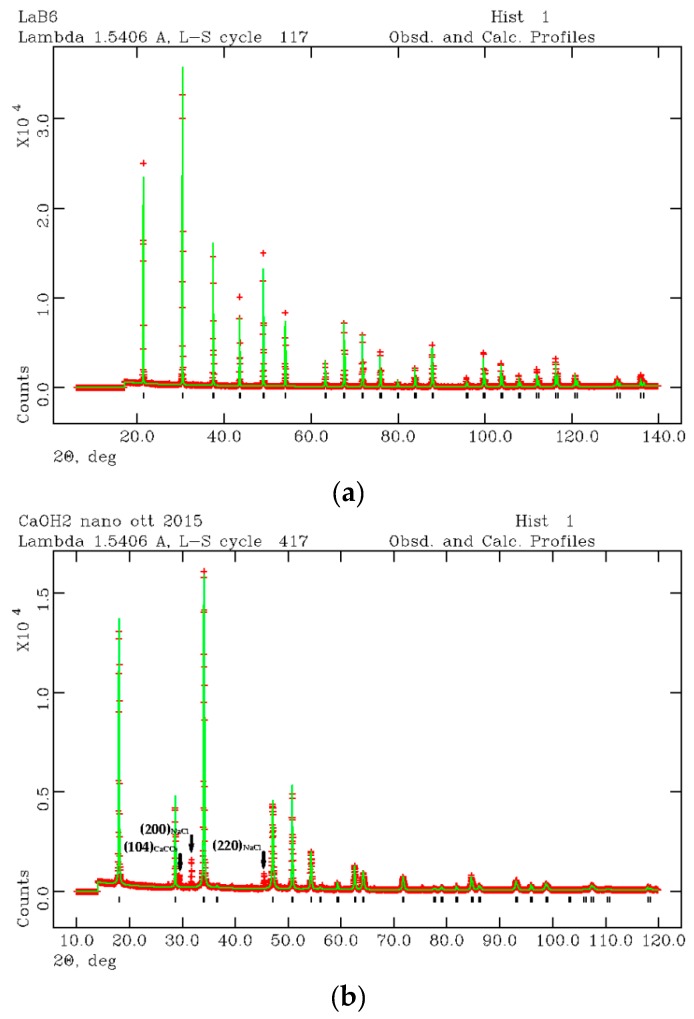
XRPD refined profiles of LaB_6_ (**a**) and nano-Ca(OH)_2_ (**b**) for microstructural analysis of the synthesized nano-Ca(OH)_2_.

BET analysis gave a specific surface area for the synthesized nano-Ca(OH)_2_ equal to 5.40 ± 0.06 m^2^/g, which corresponds, by considering round-shaped grains, to an average grain size of approximately 500 nm (diameter). In Figure 2a,b, the LaB_6_ and nano-Ca(OH)_2_ diffraction patterns are shown as refined by the Rietveld method [46]. The starting crystal structure were taken from Reference [52] and Reference [53] for Ca(OH)_2_ and LaB_6_, respectively. Results of the Rietveld refinement are reported in Tables S2 and S3 (Supplementary materials) together with the peaks position listing the (*hkl*) Miller indices (The Miller indices (*hkl*) express a family of lattice planes in the crystal structure of the mineral), the 2θ and d positions, and intensities.

The nano-Ca(OH)_2_ was found in a mixture with calcite (CaCO_3_) and halite (NaCl). In Figure 2b, three more peaks were found apart from those attributed to portlandite. The first peak is the (104) reflection of calcite (CaCO_3_) and the second and third peaks are (200) and (220) reflections of halite (NaCl). Calcite and halite are commonly present as secondary synthesis products. However, if their abundance is lower than 1 wt %, the two accessory phases were ignored from the micro-structural analysis. Thus, the microstructure analysis was performed by considering a pure Ca(OH)_2_ phase. Taking into account the (001) as the anisotropic broadening axis, the anisotropic particle size components (p) were calculated as p_parallel(001)_ = 18000Kλ/π(X + X_e_) and p_perpendicular(001)_ = 18000Kλ/πX, where K is the Scherrer constant, λ is the used wavelength, X and X_e_ coefficients are LX, and the ptec profile function are terms in GSAS [51]. Results are approximately 370 nm along (001) and 500 nm perpendicular to (001), which confirms the results obtained from BET analysis. Moreover, a strain component was calculated from the profile function refinement [28] being ~5.6 ‰ and ~1.0 ‰ along and perpendicularly to (001), respectively. This data is consistent with the hexagonal symmetry of Ca(OH)_2_ where the elongation axis is c.

### 3.2. Clinkers Analysis

**Figure 3:**
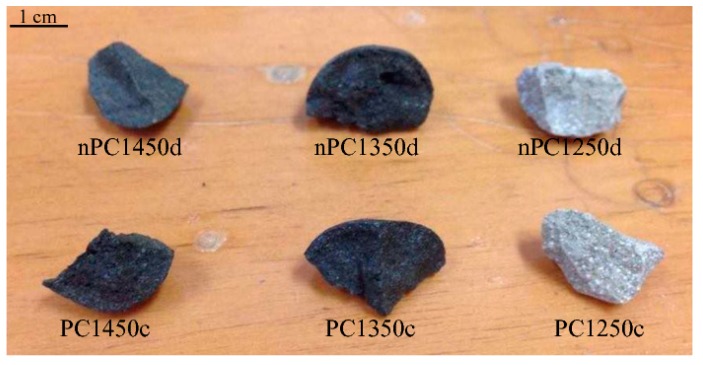
Clinkers after burning.

The obtained clinkers are shown in Figure 3. In both PC and nPC samples, colors are light grey for experiments run at 1250 °C and dark grey to blackish for experiments run at 1350 and 1450 °C, respectively. The clinker color reflects the mineralogical reactions occurring during cooking, which are completed at the highest temperature. The two populations, which are called the PC and nPC, do not show any significant macroscopic differences.

**Figure 4:**
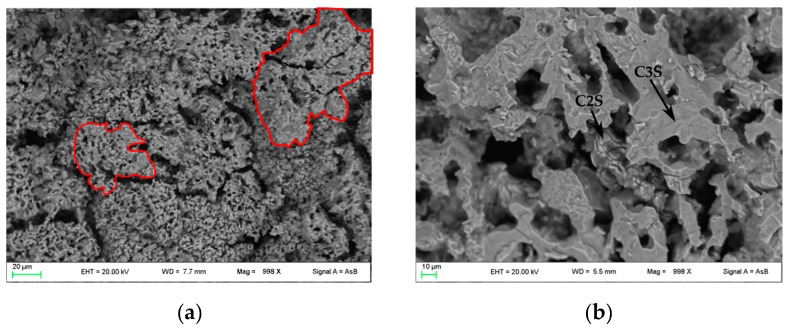

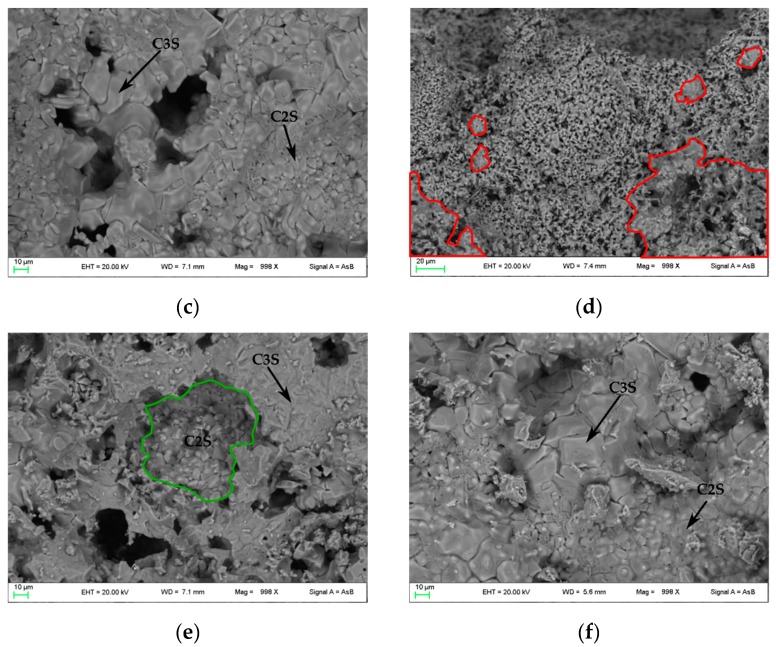
FESEM images of the clinkers (**a**) PC1250c, (**b**) PC1350c, (**c**) PC1450c, (**d**) nPC1250d, (**e**) nPC1350d, and (**f**) nPC1450d. The red areas highlight regions where mineralogical reactions started in clinkers burned at 1250 °C. The green area highlights a belite nest in nPC1350d. The C3S and C2S grains are also shown in clinkers burned at 1350 °C and 1450 °C.

In Figure 4, FE-SEM backscattered electron images are shown. In PC1250 and nPC1250, many unreacted areas are observed with a few regions where mineralogical reactions started (red areas in Figure 4a,d). Concerning clinkers burned at 1350 °C, the reacted regions increase and the clinker becomes more compact (Figure 4b,e). In nPC1350d, belite nests were also observed (green area in Figure 4e), which suggests a higher crystallinity of the sample with respect to PC1350c. Clinkers burned at 1450 °C show a sufficient textural differentiation between C3S and C2S, which have a former larger grain size with respect to the latter (Figure 4c,f). The EDS semi-quantitative chemical analyses on alite and belite give an average chemical composition of major oxides (average of 10 data points) as CaO = 77(3) wt % and SiO_2_ = 23(1) wt % for C3S, and, CaO = 68(2) wt % and SiO_2_ = 32(2) wt % for C2S.

#### 3.2.1. Clinker Porosity

**Figure 5:**
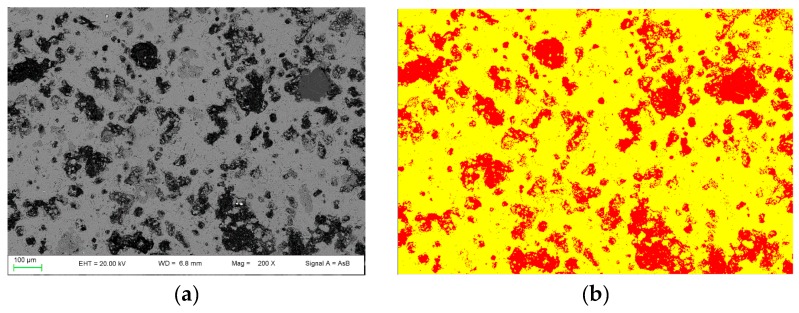

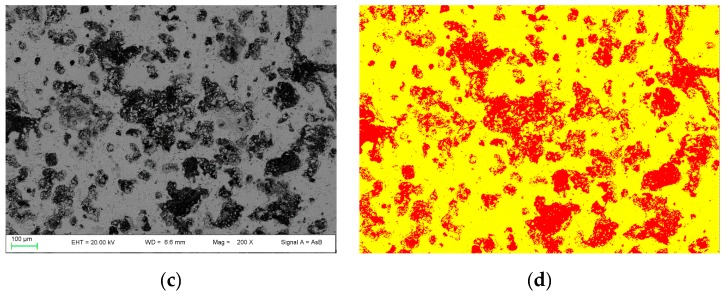
Example of FE-SEM image analysis performed on (**a**,**b**) the nPC1450d sample and (**c**,**d**) the PC1450c. The acquired images (**a**,**c**) are shown together with the processed ones (**b**,**d**).

By using the 2D image analysis method, the average porosity for samples nPC1450d and PC1450c were calculated, which is 26 ± 3 area percentage and 35 ± 2 area percentage, respectively. In Figure 5, an example of the FE-SEM image analysis procedure applied to sample nPC1450d and PC1450c is reported.

**Figure 6:**
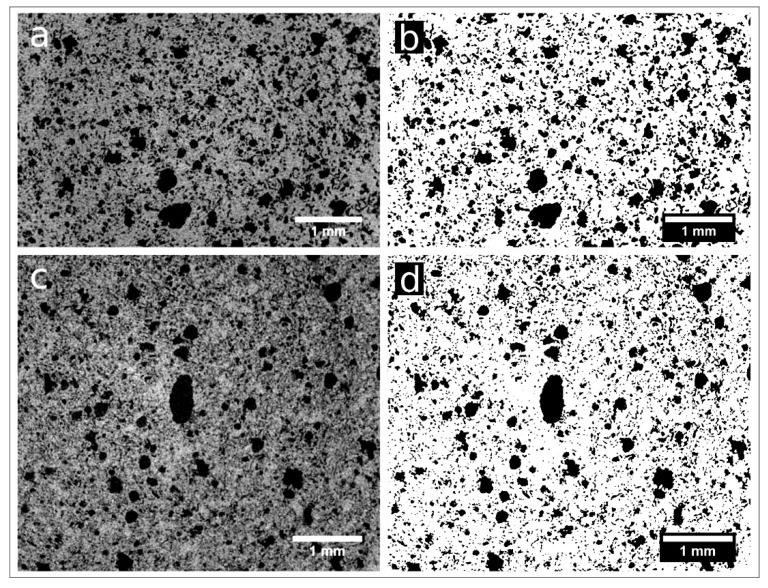
Reconstructed axial slices of samples nPC1450d (**a**) and nPC1350d (**c**) after the application of an anisotropic diffusion. (**b**,**d**) show the binary images of the same slices obtained by 3D segmentation (pores in white, background in black).

**Figure 7:**
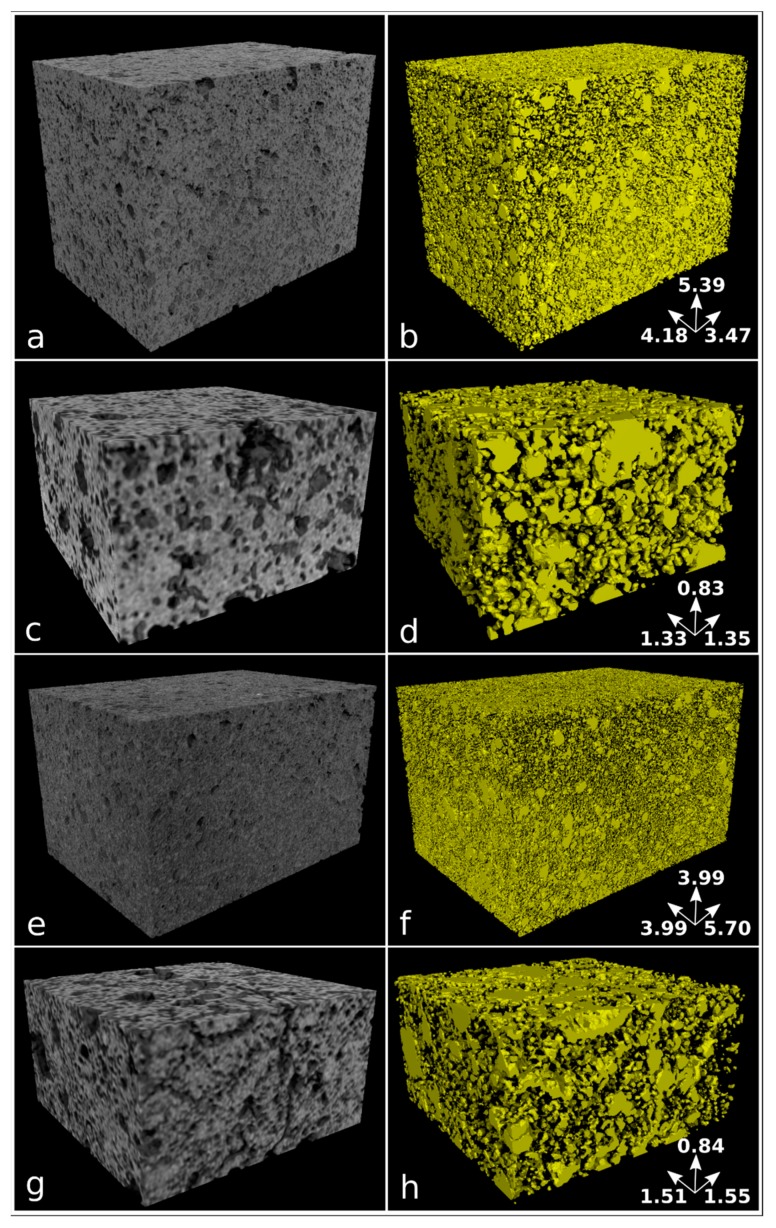
Volume renderings of the extracted VOIs from reconstructed images (left column) and corresponding iso-surface renderings of the pore phase in yellow (right column) for samples nPC1450d (**a**,**b**), PC1450c (**c**,**d**), nPC1350d (**e**,**f**), and PC1350c (**g**,**h**). Samples nPC1450d and nPC1350d have been reconstructed with an isotropic voxel size of 5.7 µm, while an isotropic voxel size of 5.0 µm has been used for samples PC1450c and PC1350c. The size of analyzed VOIs is reported in Table 2. The dimension of the sample edges is reported in mm.

Samples nPC1450d, nPC1350d, PC1450c, and PC1350c have been characterized by laboratory X-ray µCT. In Figure 6, examples of reconstructed axial slices after filtering and the corresponding binary images obtained by segmentation are reported. The whole porosities set is evident and their size qualitatively ranges from tens to several hundreds of microns. The 3D renderings of the extracted VOIs is shown in Figure 7 together with the corresponding iso-surfaces of the pore phases.

The quantitative parameters extracted by the X-ray μCT data (Figure 6 and Figure 7, Table 2) agree with observations made by the 2D analysis, only for the clinker series burned at 1350° C. The pore fraction is 0.16 and 0.23 for nPC1350d and PC1350c, respectively, which shows a decrease in the total porosity of the clinker prepared by using nano-Ca(OH)_2_. For clinkers burned at 1450 °C, the pore fraction of nPC1450d and PC1450c are comparable, being 0.24 and 0.20, respectively.

#### 3.2.2. Clinker Mineralogical Composition

Diffraction profiles of the analyzed samples and results from the Rietveld refinement of XRPD data are given as Supplementary Material in Figure S1 and Table S4, respectively. The Rietveld refinement was performed by taking into account polymorphs of each clinker mineral phase. The starting structural parameters were taken for cubic C3A from Reference [54], for C2S-γ from Reference [55], for C2S-β from Reference [56], for monoclinic C3S from References [57] and [58], for triclinic C3S from Reference [59], for C4AF from Reference [60], for ghelenite from Reference [61], for CaO from Reference [62], and for Ca(OH)_2_ from Reference [52]. Since the analysis of polymorphs abundance was not an objective of the present work, polymorphs of the same phase were summed up by giving the total abundance of the mineral. With regard to the free lime, since CaO is a strong hydrophilic material, we considered Ca(OH)_2_ as a secondary product coming from the CaO hydration at ambient conditions. Thus, CaO and Ca(OH)_2_ were summed up by giving the total amount of free lime after clinker burning.

Results from the Rietveld refinement of XRPD data (Table S4 as Supplementary Material) showed that no correlation of the amorphous content is observed neither with experimental temperatures nor with the nano-Ca(OH)_2_ addition. The spread distribution of the amorphous content might be due to the quenching rate, likely being the amorphous formation very sensitive to this parameter. Thus, further effort is needed to check for the amorphous formation conditions and to set up an experimental procedure suitable to study the amorphous content behavior as a function of clinker burning T and nano-material addition. For these reasons, data were treated in the present work without considering the amorphous content and they were normalized to the crystalline portion of the samples (Table 3).

**Figure 8:**
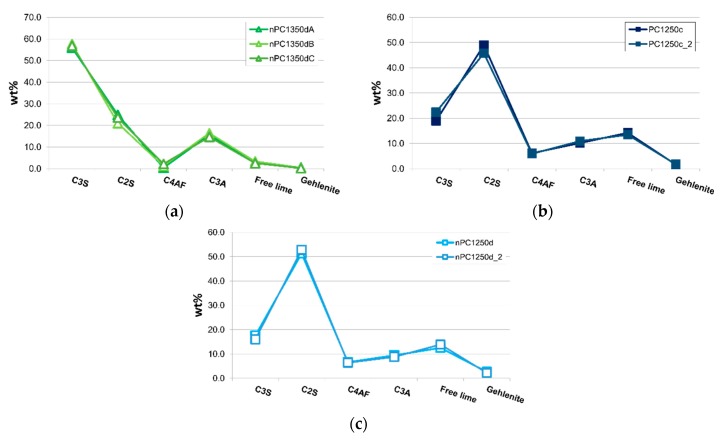
Spider diagrams of (**a**) nPC1350dA, nPC1350dB, and nPC1350dC and (**b**) PC1250c and PC1250c_2 and (**c**) nPC1250d and nPC1250d_2. Error bars in wt % are within the symbols. The CaO + Ca(OH)_2_ content is expressed as “Free lime.”

In Figure 8a, samples nPC1350dA, nPC1350dB, and nPC1350dC are shown. The three samples are different portions of the same run product (nPC1350d). A very good overlapping of the three data distributions confirms the homogeneity within the clinker samples. Data from the three repeated experiments were then averaged. Checking for the reproducibility of the experimental set up, two different portions of the same raw mixture were fired at the same conditions for PC1250c and nPC1250d. The obtained clinkers PC1250c and PC1250c_2 were compared (Figure 8b) as well as nPC1250d and nPC1250d_2 (Figure 8c). Again, a very good reproducibility is shown, so the data from the previously mentioned repetitions were averaged in pair.

## 4. Discussion

The 2D FE-SEM study of the porosity features of the synthesized clinkers showed a decrease in the total porosity fraction of the clinkers produced by nano-Ca(OH)_2_. The same observation was made by the X-ray µ-CT analyses for the clinker burned at 1350 °C, which has values for the total porosity fraction of 0.23 and 0.16 for PC1350c and nPC1350d, respectively. Concerning the clinkers burned at 1450 °C, the tomographic analysis showed that PC1450c and nPC1450d have close values of total porosity fraction being 0.20 and 0.24, by volume, respectively.

**Figure 9:**
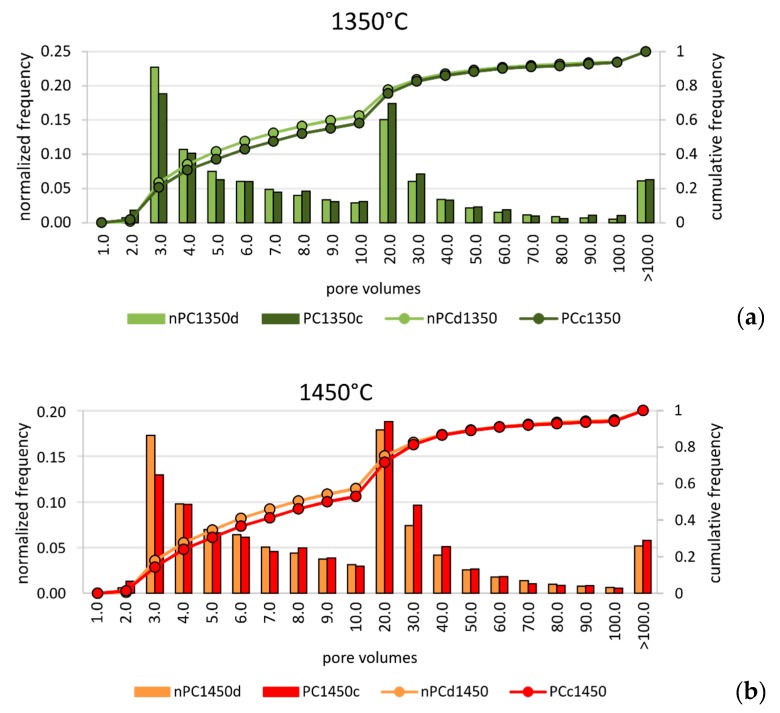
Pore size distribution of volumes for (**a**) nPC1350d - PC1350c and (**b**) nPC1450d - PC1450c. Both normalized frequencies (bars) and cumulative frequencies (lines) are given. The observations were grouped in 20 classes, as shown in the abscissa. Units are 10^−6^ mm^3^.

In order to discriminate possible microstructural differences in the clinkers, a pore size distribution analysis was performed. Since the analyzed clinker volumes were not the same in the four samples, data were normalized to the total number of pores for each sample. Results are given in Figure 9. In the normalized pore size distribution of both nano-added clinkers and standard Portland clinkers, two main modal distributions were observed: the first group with tiny porosity (hereafter “tiny porosity”) was characterized by a modal volume that corresponds to the class 3 × 10^−6^ mm^3^. The second group with modal volume lied in the class 2 × 10^−5^ mm^3^ (hereafter “large porosity”). Nonetheless, the similarity in the modal distribution of the analyzed samples at each temperature, showed intriguing differences when nano-Ca(OH)_2_ was used. In fact, the nano-added clinkers have higher frequencies in the “tiny porosity” range with respect to the standard Portland clinkers, particularly for the class 3.0 × 10^−6^ mm^3^ (Figure 9). The “large porosity”, on the contrary, showed the opposite behavior, by decreasing in the nano-added clinkers even if its variation is less evident than the “tiny porosity” increase.

Notwithstanding the higher resolution of FE-SEM instrument with respect to X-ray μCT, the amount of “tiny porosity” in nPC1450d is likely underestimated by the former. This behavior might arise because of the non-representativeness of single FE-SEM 2D images with respect to the 3D volumes obtained by the X-ray μCT technique. The overestimation was particularly important for the nPC1450d sample, where the increase of the “tiny porosity”, with respect to PC1450c, was more evident than in the 1350 °C clinkers series (Figure 9).

Many studies dealt with the effect of porosity and pore size distribution on materials’ grindability [63,64,65,66]. In general, the strength of a porous medium increases as (i) porosity decreases, (ii) small pores increase, and (iii) large pores decrease. Thus, the porosity analysis performed, in this case, suggested that the clinkers produced by using nano-Ca(OH)_2_ had higher strength than the standard Portland clinkers, which might reflect, in turn, higher energy consumption during clinker grinding.

**Figure 10:**
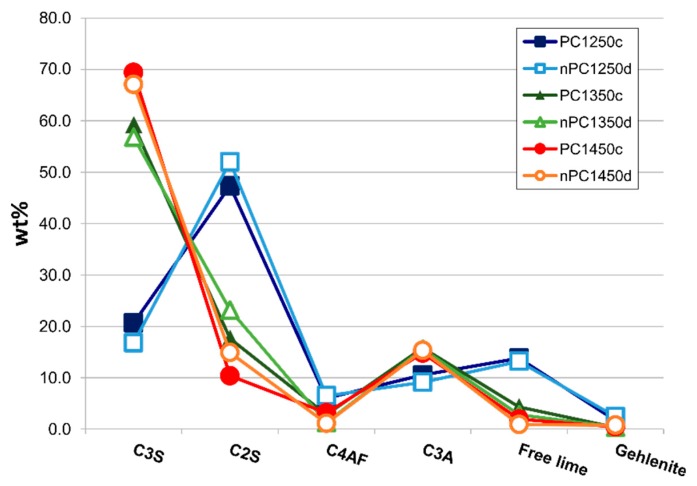
Spider diagram for the mineralogical quantitative analysis (Rietveld refinement Reference [46]) for the whole data set. Error bars in wt % are within the symbols.

In Figure 10, the mineralogical compositions of the “cooked” clinkers are compared. Samples are divided in two main groups: (i) clinkers “cooked” at 1250 °C with low C3S and high C2S and free lime content reflecting that the mineralogical reactions are not completed (i.e., C3S has not been formed yet), and (ii) clinkers “cooked” at 1350 and 1450 °C with higher C3S abundance in standard Portland clinkers.

**Figure 11:**
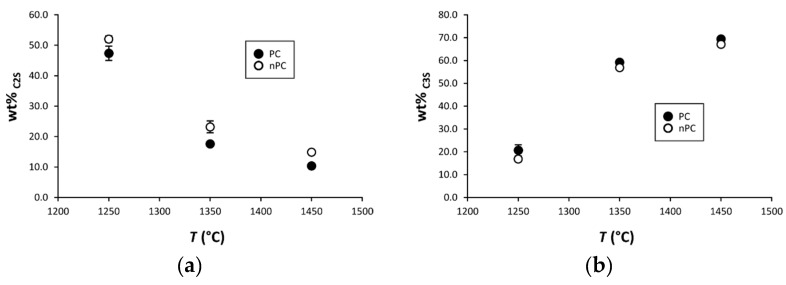
Binary plots (**a**) C2S vs. *T* and (**b**) C3S vs. *T* for PC (filled symbols) and nPC (empty symbols) at the three burning temperatures. If not present, error bars in wt % are within the symbols.

**Figure 12:**
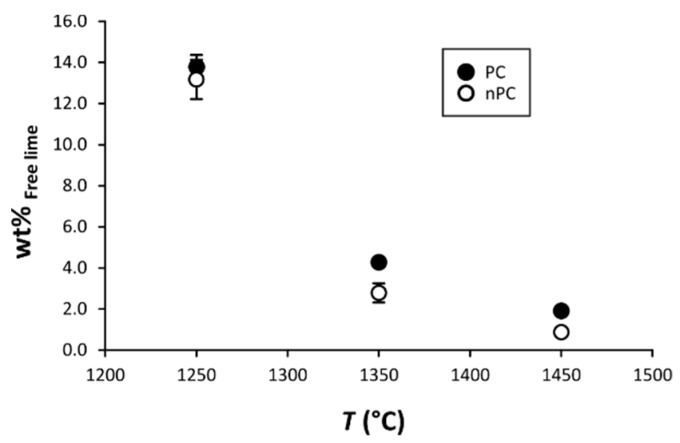
Binary plot free lime vs. *T*. The free lime content after clinker burning is given as CaO + Ca(OH)_2_. If not present, error bars in wt % are within the symbols.

By the addition of the nano-Ca(OH)_2_, modifications on the clinkers’ mineralogical composition occurred (Figure 10, Figure 11 and Figure 12). In particular, the use of nano-Ca(OH)_2_ in the raw mixture led to a decrease of free lime in the final product (Figure 12), especially for clinkers burned at 1350 °C and 1450 °C. In fact, for PC1250c and nPC1250d, the free lime content is 13.8(6) wt % and 13(1) wt %, respectively. However, free lime is 4.28(2) wt % in PC1350c and 2.8(5) wt % in nPC1350d, and, 1.92(1) wt % in PC1450c and 0.87(1) wt % in nPC1450d. The evolution of the free lime content in a clinker is of paramount importance to give constraints on the clinker burnability. The observed decrease in the free lime content in nPC with respect to PC at all of the three burning temperatures is related to an improvement of the nano-added clinkers’ cooking efficiency. Additionally, the clinkers produced by using nano-Ca(OH)_2_ show a slight increase of C2S (approximately 2–4 wt %) and decrease of C3S (approximately 5–6 wt %), which is also shown in Figure 11. These variations are consequences of the higher reactivity of nPC with respect to PC.

The reason for the higher reactivity of nPC with respect to PC is due to the lower diffusion activation energy of nano-CaO with respect to the higher-sized materials. The complete reaction of nano-CaO during the first burning stage, where C2S formation is the dominant reaction, (1) increases the number of C2S regions within the clinker grains and (2) prevents the C3S reaction kinetic to be affected by the enhancing Ca^2+^ diffusion due to the nano-Ca(OH)_2_ addition. However, the value of C3S in nPC1350d and nPC1450d, which is approximately 56.8(8) wt % and 67.1(1) wt %, respectively, lies in range of C3S optimal amount for standard Portland clinkers (50–70 wt % [4]).

Regarding the behavior of minor phases, a C4AF decrease in the nPC samples at 1350 °C and 1450 °C burning temperatures was observed, being at 1350 °C C4AF = 2.8(1) wt % in PC and C4AF = 1.2(1) wt % in nPC, at 1450 °C C4AF = 3.1(1) wt % in PC and C4AF = 1.0(1) wt % in nPC. The C3A content, on the contrary, does not have significant variations among the PC and nPC samples. The C4AF and C3A content are important parameters since they are related, together with silicates, to the work index of the clinkers that measures the energy needed for clinker grinding [67,68]. In particular, the higher the ratio between C3S + C2S and C4AF + C3A, the lower the work index. Results from the present research gives C3S + C2S / C4AF + C3A equal to: 4.13(3) and 4.77(2) for PC1350c and nPC1350d, respectively, as well as 4.45(2) and 4.99(1) for PC1450c and nPC1450d, respectively. The nPC clinkers have a lower ratio C3S + C2S / C4AF + C3A, which reflects a decrease of the energy required for clinker grinding. Data of samples PC1250c and nPC1250d cannot be compared to 1350 °C and 1450 °C clinkers. In fact, at *T* < 1300 °C, no melting occurs and the C4AF phase forms at sub-solidus conditions between C3A and other Ca and Fe oxides. The enhanced Ca diffusion in nPC raw mixtures might lead to a faster C3A formation, while C3A formation is directly dependent on Ca diffusion into Al_2_O_3_ [69], which is available to react with CF producing C4AF. Thus, the C4AF formation reaction might be fastened as well. As such, in nPC1250d, we expect a decrease in C3A and an increase in C4AF. Data confirm such a hypothesis, being C3A = 10.51(1) wt % in PC1250c and C3A = 9.10(1) wt % in nPC1250d, whereas, C4AF = 6.00(1) wt % in PC1250c and C4AF = 6.49(1) wt % in nPC1250d.

The present research pointed out that the use of nano-Ca(OH)_2_ as raw material for clinker production, in replacement of part of the carbonates, is a suitable technique to enhance the clinker cooking efficiency. This might reflect, in turn, the improvement of the sustainability of the clinker production process, in three main aspects. The first one is related to the reduction of the CO_2_ process emission as Ca(OH)_2_, which is already decarbonated. This was used instead of CaCO_3_. For each ton of Ca(OH)_2_ used in the raw meal, we can save 0.595 ton of CO_2_. The second contribution is connected to the decrease of the required energy for clinker burning due to the increase of the cooking efficiency of the raw meal. The upper limit on the rate of kiln feed is due to the heat energy that could be produced in the kiln by the combustion process. Therefore, if we decrease the required energy for each kilos of raw meal, we could also use the same total energy produced inside the kiln to increase the plant feeding rate and, in turn, increase the plant productivity. The third aspect is related to the grinding process. The increase of the silicates to the aluminate ratio in nPC clinkers led to a decrease in the grinding energy requirement. This contribution is of paramount importance because it might limit the effect of the mechanical strength increase in nPC with respect to PC due to the evolution of the pore size distribution.

## 5. Conclusions

In this study, we tested a new generation of clinkers produced by the substitution of part of the carbonate component by nano-Ca(OH)_2_. The aim was to improve the sustainability of the Portland clinker production process by increasing the cooking efficiency of the raw mixtures.

Results showed that, at 1250 °C, the alite formation reaction has not started yet in both PC and nPC raw mixtures. By increasing the temperature to 1350 and 1450 °C, C3S is formed in both PC and nPC. The nano-addition leads to the free lime decrease in the final products especially for clinkers burned at 1350 and 1450 °C. This confirms the increase of the nPC raw mixtures’ cooking efficiency. The relatively low energy required for the clinker firing could be used to increase the plant productivity and decrease the CO_2_ emissions during clinker burning. The decrease of the work index of the clinkers produced by the use of the nano-Ca(OH)_2_ also contributes to the energy saving during clinker grinding. However, a Life Cycle Analysis (LCA) for the entire process might be the key to quantify the energy gain/loss for each production step and for the whole process.

As for the porosity analysis of the synthesized samples, the 3D analytical approach gives more reliable results of the volumetric pore size distribution within the macroscopic volumes compared to the 2D analysis. In addition, despite the lower spatial resolution, 3D images obtained by microtomography are more representative of the samples than the 2D images collected by FE-SEM 2D. The nano-added clinkers burned at 1350°C showed a decrease in the total porosity with respect to the standard Portland clinkers having a very abundant population of tiny porosities in the range of 3.0 to 7.0 × 10^−6^ mm^3^.

Lastly, future studies will be devoted to check for possible solutions, such as minor and trace elements addition to the raw mixtures, in order to enhance the C3S formation in the nano-added clinkers, even if its abundance already lies in the range for the standard Portland clinkers. 

## Figures and Tables

**Table 1:** Chemical composition and Loss on Ignition (LOI) (wt %) of marls and raw mixtures. PC is the standard Portland Clinker and nPC is the Portland Clinker produced by using 5 wt % of nano-Ca(OH)_2_ in the raw mixture. The LSF (Lime Saturation Factor), SR (Silica Ratio), and AR (Alumina Ratio) parameters [4] are also listed.

Material	Na_2_O	MgO	Al_2_O_3_	SiO_2_	K_2_O	CaO	Fe_2_O_3_	LOI	LSF	SR	AR
**“Fat marl”**	0.101	0.928	3.511	12.098	0.918	44.627	1.241	36.576			
**“Slim marl”**	0.091	1.056	6.610	23.101	1.258	35.315	2.314	30.255			
**PC**	0.100	0.945	3.914	13.528	0.962	43.417	1.381	35.754	2.5550	2.8352	0.9987
**nPC**	0.094	0.909	3.983	13.792	0.943	44.234	1.403	34.642	2.5605	2.8387	0.9983

**Table 2:** Results obtained by applying the Basic Analysis module of the Pore3D software library extracted from the laboratory X-ray μCT data.

Sample	Volume (voxels)	Voxel Size (µm)	Volume (mm^3^)	Pores Thresholds	Eulerian Characteristics (mm^−3^)	Internal Mean Curvature (mm^−2^)	Specific Surface Area (mm^−1^)	Pore Fraction
nPC1450d	946 × 610 × 734	5.7	52.95	91	573	712	22.51	0.24
PC1450c	271 × 267 × 167	5.0	1.51	93	885	711	20.72	0.20
nPC1350d	700 × 700 × 1000	5.7	61.25	91	2048	711	16.77	0.16
PC1350c	303 × 168 × 311	5.0	1.98	76	1013	560	18.09	0.23

**Table 3:** Results of Rietveld refinement of XRPD data [46]. Data are normalized to the crystalline portion of the analyzed samples, without taking into account the amorphous content.

Sample	C3S	C2S	C4AF	C3A	Free Lime	Gehlenite
PC1250c	18.92(6)	49.0(1)	6.13(2)	10.14(2)	14.20(4)	1.61(1)
PC1250c_2	22.37(2)	45.71(2)	5.85(1)	10.92(1)	13.40(1)	1.76(1)
PC1350c	59.2(2)	17.61(7)	2.81(1)	15.80(5)	4.28(2)	0.31(1)
PC1450c	69.4(1)	10.38(2)	3.15(1)	14.77(3)	1.92(3)	0.39(1)
nPC1250d	17.56(2)	51.29(4)	6.59(1)	9.43(1)	12.50(1)	2.64(1)
nPC1250d_2	16.00(2)	52.77(4)	6.39(1)	8.78(1)	13.85(1)	2.21(1)
nPC1350dA	56.82(6)	23.63(2)	2.19(1)	14.66(1)	2.50(1)	0.20(1)
nPC1350dB	56.0(2)	24.9(1)	0.42(1)	15.64(6)	2.53(1)	0.55(1)
nPC1350dC	57.66(7)	21.03(2)	1.18(1)	16.39(2)	3.33(1)	0.41(1)
nPC1450d	67.06(5)	14.88(1)	1.12(1)	15.31(1)	0.88(1)	0.74(1)

## Supplementary Materials

The following are available online at https://www.mdpi.com/1996-1944/12/11/1787/s1. Figure S1: XRPD patterns refined by means of the Rietveld method [46]. Table S1: Experimental parameters used for the laboratory X-ray microtomography measurements. Table S2: Results of the Rietveld refinement of LaB_6_. Table S3: Results of the Rietveld refinement of Ca(OH)_2_. Table S4: Results from XRPD data refinement by the Rietveld method.
