# Supplementary material for: Effect of the Nano-Ca(OH)2 Addition on the Portland Clinker Cooking Efficiency
Source: Materials (Basel). 2019 Jun 2;12(11):1787. doi: 10.3390/ma12111787 (PMC6600755; doi:10.3390/ma12111787)
Supplement: Supplementary file 1 [file materials-12-01787-s001.pdf]

materials

Supplementary Materials

Article

Effect of the Nano-$Ca(OH)_2$ Addition on the Portland Clinker Cooking Efficiency

Azzurra Zucchini [1,*], Paola Comodi [1], Alessandro Di Michele [1], Riccardo Vivani [2], Lucia Mancini [3], Gabriele Lanzafame [3], Serena Casagrande [2], Silvia Gentili [1], Francesco Vetere [1,5], Luca Bartolucci [1], Gianluca Polidori [1], Fabio Santinelli [4] and Alessandro Neri [4]

[1] Department of Physics and Geology, University of Perugia, Via Pascoli, 06123 Perugia, Italy; paola.comodi@unipg.it (P.C.); alessandro.dimichele@unipg.it (A.D.M.); silvia.gentili85@gmail.com (S.G.); francesco.vetere@unipg.it (F.V.); luca.bartolucci@unipg.it (L.B.); gianluca.polidori@unipg.it (G.P.)
[2] Department of Pharmaceutical Sciences, University of Perugia, Via A. Fabbretti, 06123 Perugia, Italy; riccardo.vivani@unipg.it (R.V.); serena.casagrande@studenti.unipg.it (S.C.)
[3] Elettra - Sincrotrone Trieste S.C.p.A., SS 14, Km 163.5 in Area Science Park, 34149 Basovizza (Trieste), Italy; lucia.mancini@elettra.eu (L.M.); gabriele.lanzafame@gmail.com (G.L.)
[4] Colacem S.p.A., Via della Vittorina, 06024, Gubbio, Italy; f.santinelli@financo.it (F.S.); a.neri@financo.it (A.N.)
[5] Institut of Mineralogy, Leibniz Universität Hannover, Callinstrasse 3, 30167 Hannover, Germany
* Correspondence: azzurra.zucchini@unipg.it

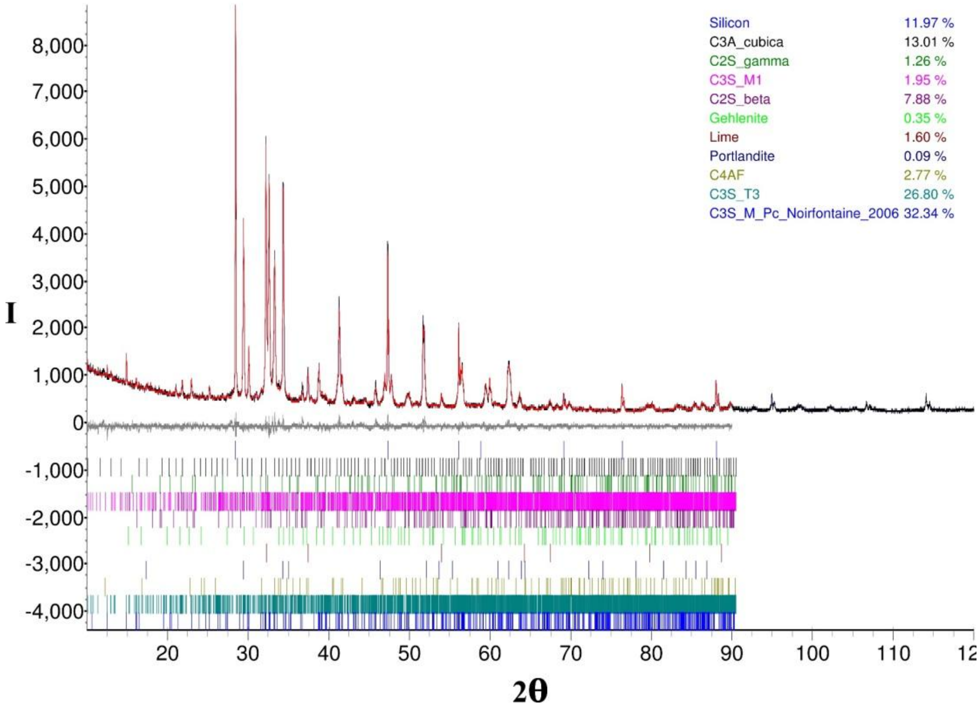

(a)

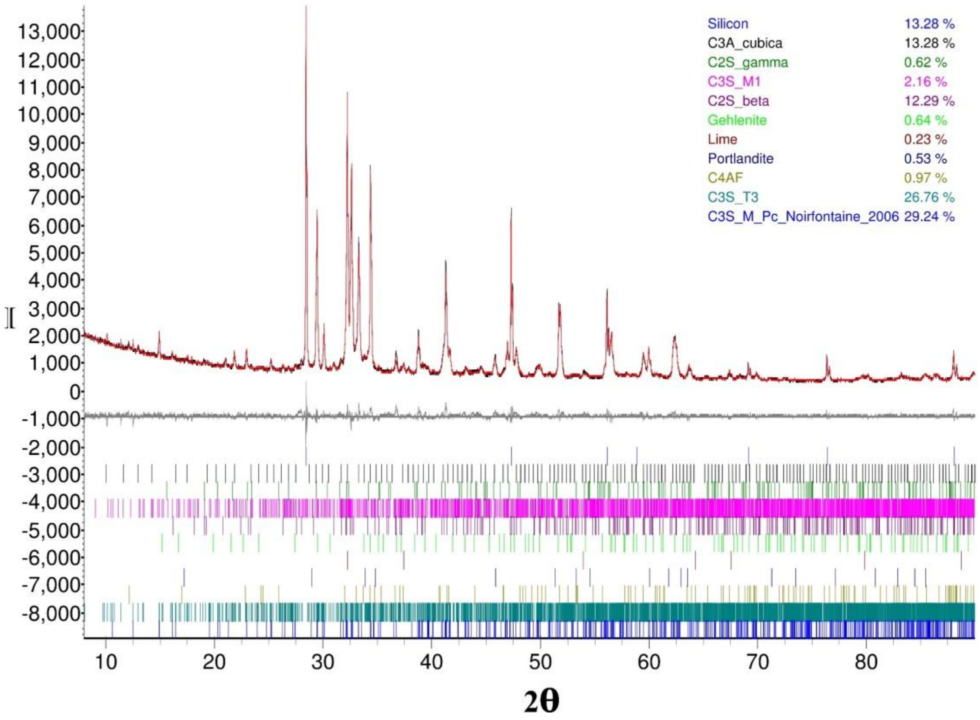
Silicon 13.28 %
C3A_cubica 13.28 %
C2S_gamma 0.62 %
C3S_M1 2.16 %
C2S_beta 12.29 %
Gehlenite 0.64 %
Lime 0.23 %
Portlandite 0.53 %
C4AF 0.97 %
C3S_T3 26.76 %
C3S_M_Pc_Noirfontaine_2006 29.24 %
I
2θ

(b)

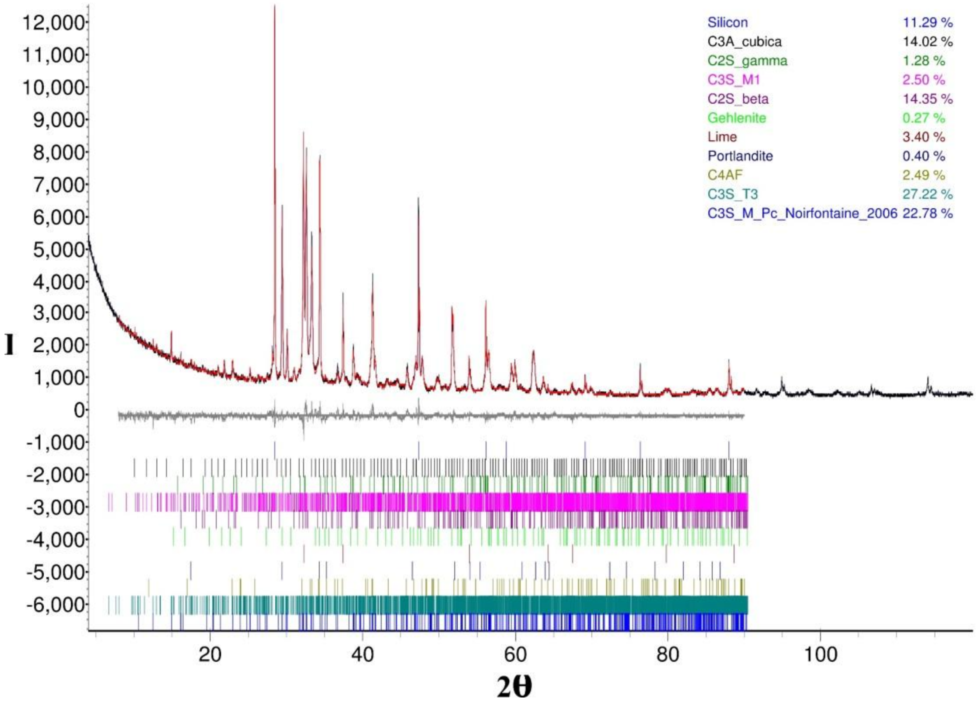
Silicon 11.29 %
C3A_cubica 14.02 %
C2S_gamma 1.28 %
C3S_M1 2.50 %
C2S_beta 14.35 %
Gehlenite 0.27 %
Lime 3.40 %
Portlandite 0.40 %
C4AF 2.49 %
C3S_T3 27.22 %
C3S_M_Pc_Noirfontaine_2006 22.78 %
I
2θ

(c)

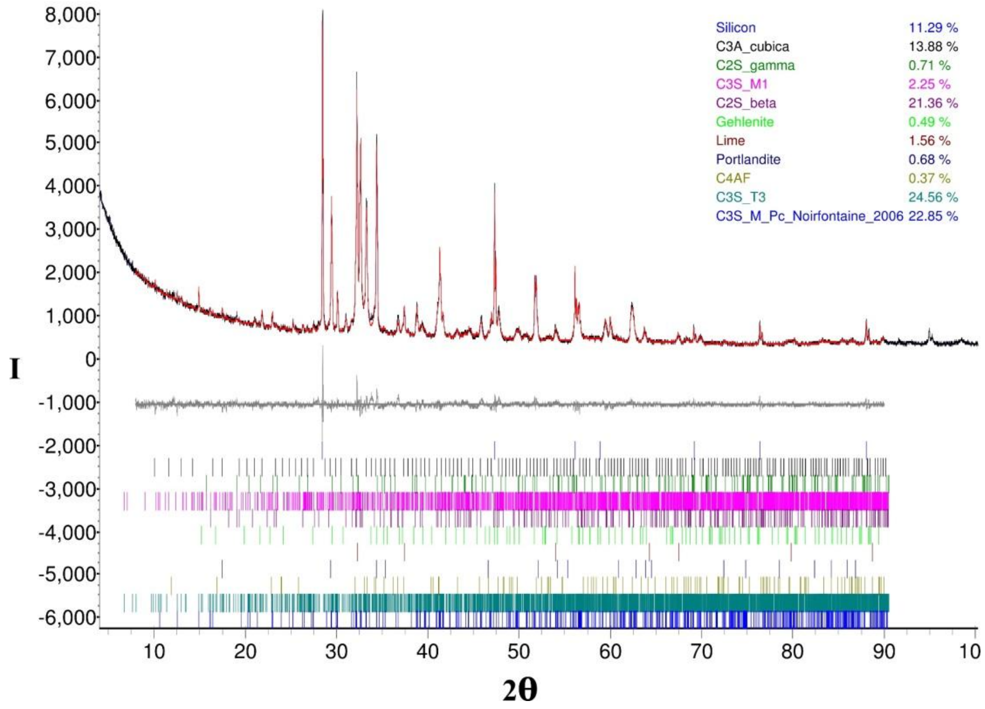
Silicon 11.29 %
C3A_cubica 13.88 %
C2S_gamma 0.71 %
C3S_M1 2.25 %
C2S_beta 21.36 %
Gehlenite 0.49 %
Lime 1.56 %
Portlandite 0.68 %
C4AF 0.37 %
C3S_T3 24.56 %
C3S_M_Pc_Noirfontaine_2006 22.85 %
I
2θ

(d)

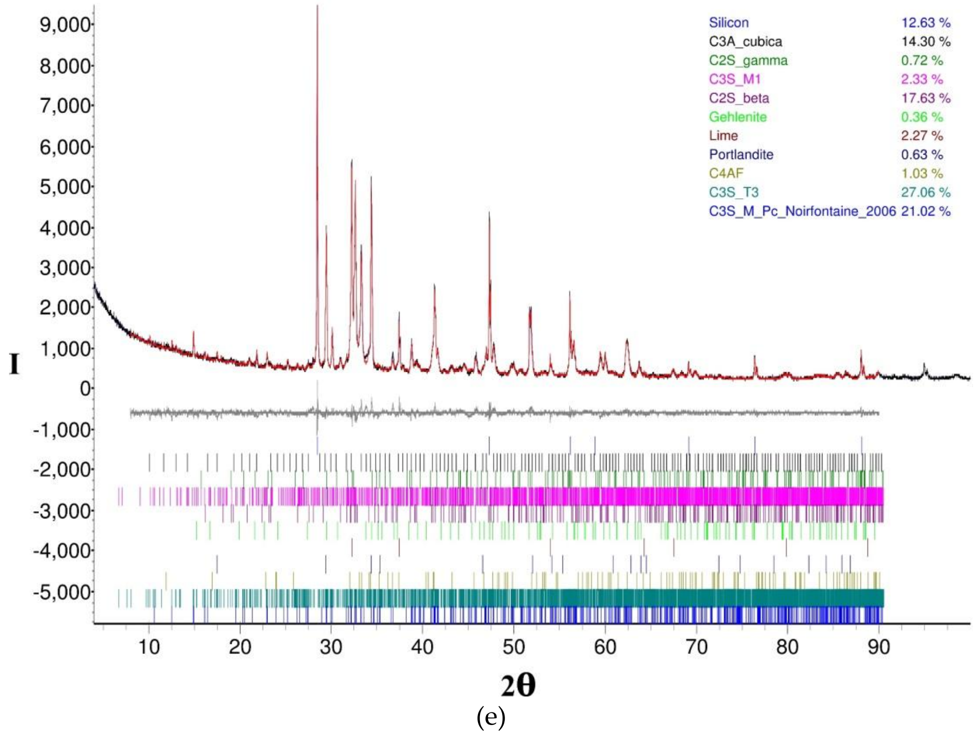
Silicon 12.63 %
C3A_cubica 14.30 %
C2S_gamma 0.72 %
C3S_M1 2.33 %
C2S_beta 17.63 %
Gehlenite 0.36 %
Lime 2.27 %
Portlandite 0.63 %
C4AF 1.03 %
C3S_T3 27.06 %
C3S_M_Pc_Noirfontaine_2006 21.02 %
I
2θ

(e)

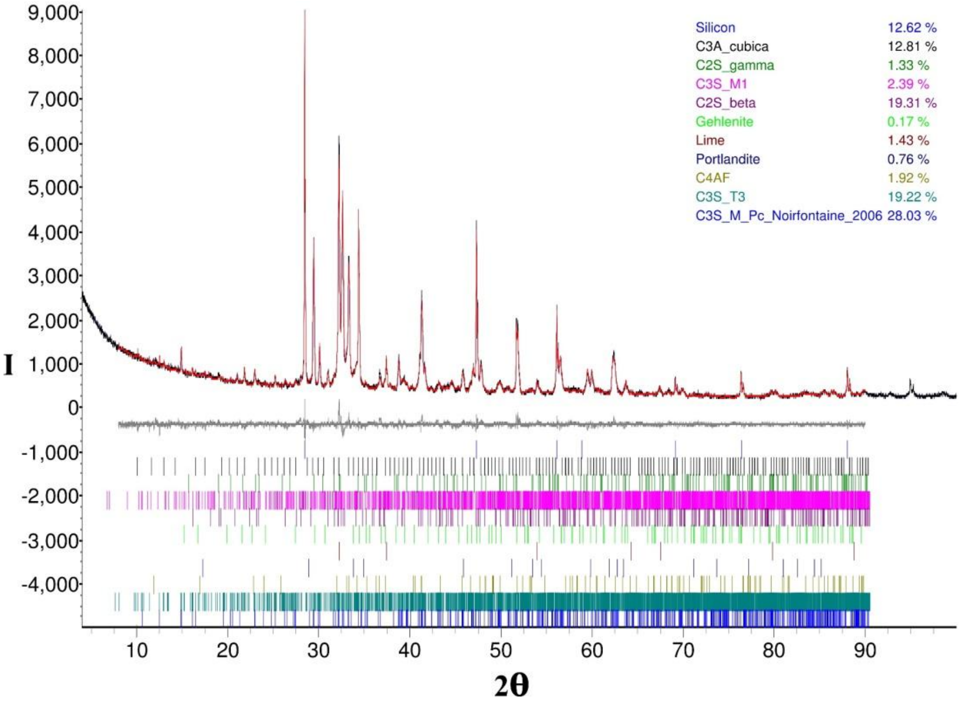

Silicon 12.62 %
C3A_cubica 12.81 %
C2S_gamma 1.33 %
C3S_M1 2.39 %
C2S_beta 19.31 %
Gehlenite 0.17 %
Lime 1.43 %
Portlandite 0.76 %
C4AF 1.92 %
C3S_T3 19.22 %
C3S_M_Pc_Noirfontaine_2006 28.03 %
I
2θ

(f)

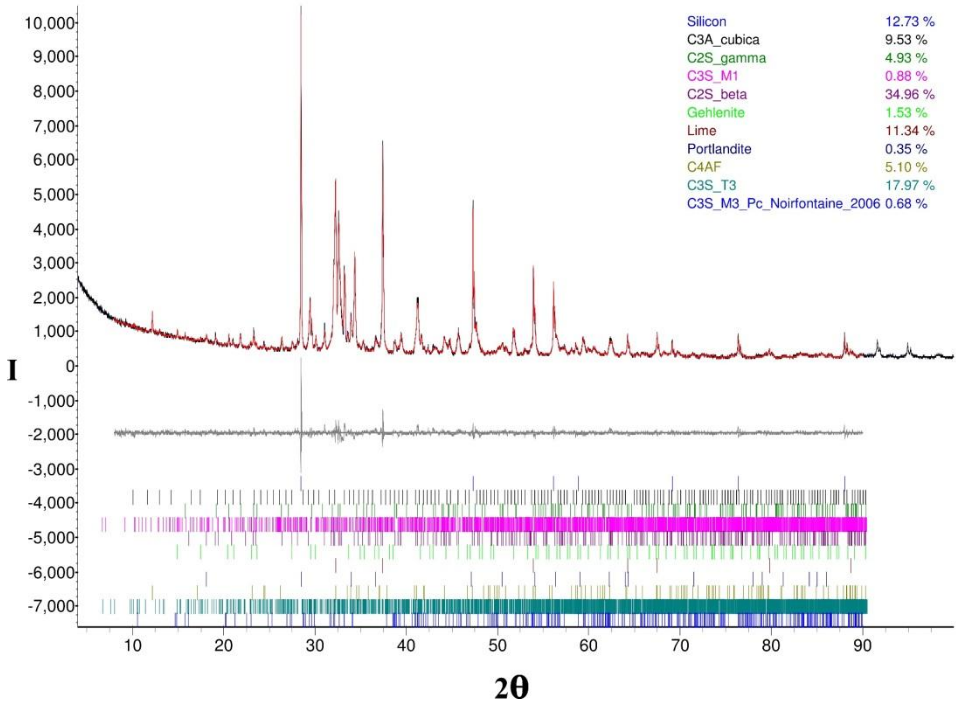

Silicon 12.73 %
C3A_cubica 9.53 %
C2S_gamma 4.93 %
C3S_M1 0.88 %
C2S_beta 34.96 %
Gehlenite 1.53 %
Lime 11.34 %
Portlandite 0.35 %
C4AF 5.10 %
C3S_T3 17.97 %
C3S_M3_Pc_Noirfontaine_2006 0.68 %
I
2θ

(g)

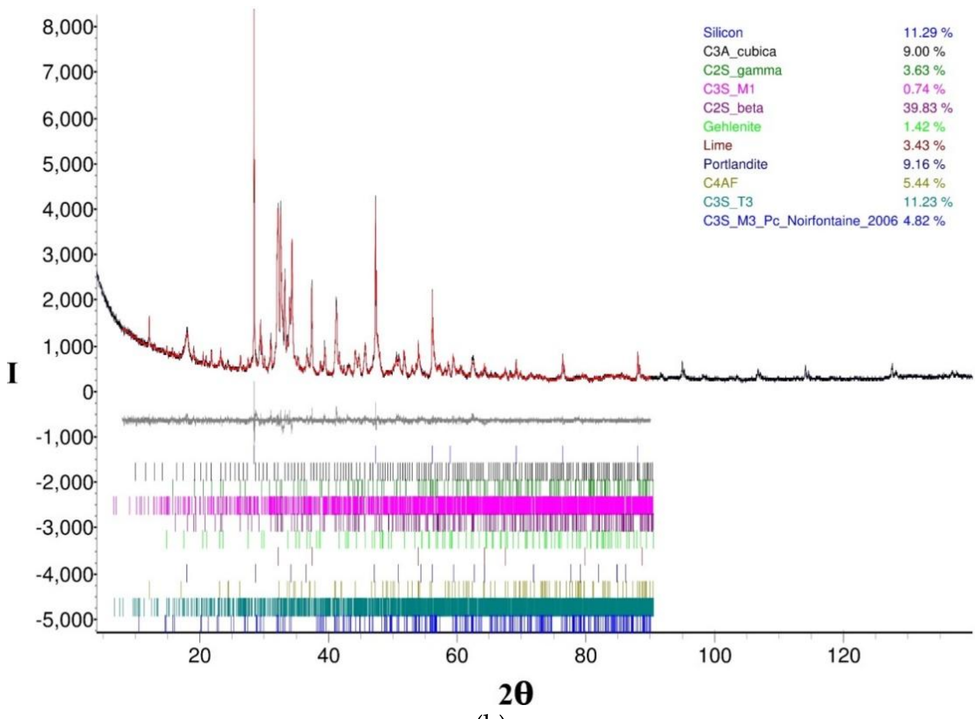

Silicon 11.29 %
C3A_cubica 9.00 %
C2S_gamma 3.63 %
C3S_M1 0.74 %
C2S_beta 39.83 %
Gehlenite 1.42 %
Lime 3.43 %
Portlandite 9.16 %
C4AF 5.44 %
C3S_T3 11.23 %
C3S_M3_Pc_Noirfontaine_2006 4.82 %
I
2θ

(h)

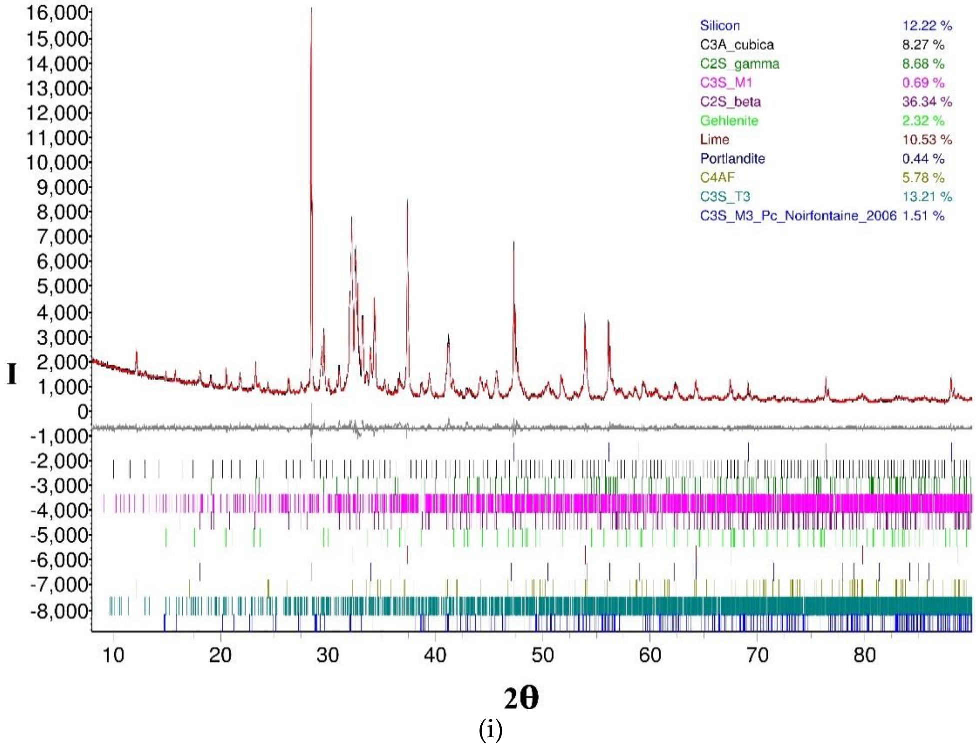

Silicon 12.22 %
C3A_cubica 8.27 %
C2S_gamma 8.68 %
C3S_M1 0.69 %
C2S_beta 36.34 %
Gehlenite 2.32 %
Lime 10.53 %
Portlandite 0.44 %
C4AF 5.78 %
C3S_T3 13.21 %
C3S_M3_Pc_Noirfontaine_2006 1.51 %
I
2θ

(i)

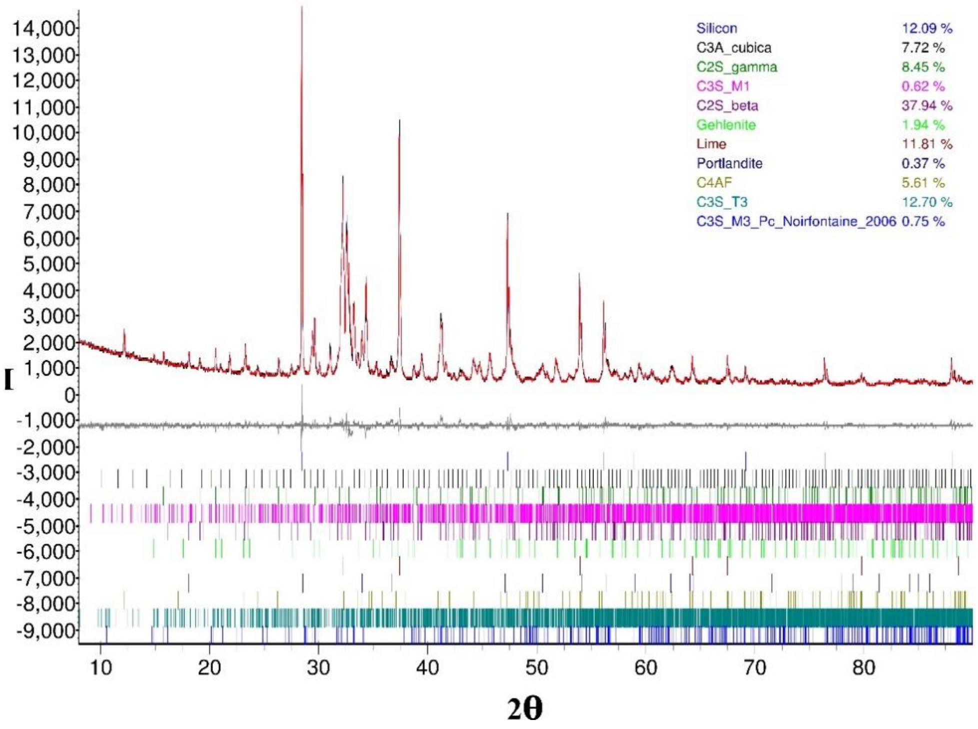

(j)

Figure S1. XRPD patterns refined by means of the Rietveld method [46]. (**a**) PC1450c; (**b**) nPC1450d; (**c**) PC1350c; (**d**) nPC1350dA; (**e**) nPC1350dB; (**f**) nPC1350dC; (**g**) PC1250c; (**h**) PC1250c_2; (**i**) nPC1250d; (**j**) nPC1250d_2.

Table S1. Experimental parameters used for the laboratory X-ray microtomography measurements.

Sample	d_1 (mm)	d_2 (mm)	Voltage (kV)	Current (μA)	Isotropic Voxel Size (μm)	Number of Projections Over 360°	Al Filter Thickness (mm)	Exposure Time (s)
nPC1450d	80	350	130	61	5.7	2400	1.50	5.0
PC1450c	80	400	90	89	5.0	2400	0.75	7.0
nPC1350d	80	350	130	61	5.7	2400	1.50	5.0
PC1350c	80	400	90	89	5.0	2400	0.75	7.0

d_1 = source-to-sample distance; d_2 = source-to-detector distance

Table S2. Results of the Rietveld refinement of LaB_6. Cell parameters (Å), cell volume (Å^3) and refined atomic positions are given together with the agreement parameters Rp and Rwp. The peaks position listing the (*hkl*) Miller indices, the 2θ(°) and *d* positions (Å), and relative intensities normalized to 100 (I) are also given.

Unit Cell Parameters		Structure Refinement			Diffraction Data			
a	*V*	x_B	Rp	Rwp	h k l	2θ	*d*	I
4.1567(1)	71.821(1)	0.1895(8)	0.089	0.114	1 0 0	21.36	4.1567	68.99
					1 1 0	30.39	2.9392	100.00
					1 1 1	37.44	2.3999	42.92
					2 0 0	43.51	2.0784	28.37
					2 1 0	48.96	1.8589	55.40

2 1 1	53.99	1.6970	29.74
2 2 0	63.22	1.4696	10.61
3 0 0	67.55	1.3856	2.78
2 2 1	67.55	1.3856	28.30
3 1 0	71.75	1.3145	23.07
3 1 1	75.85	1.2533	16.12
2 2 2	79.88	1.1999	2.58
3 2 0	83.85	1.1529	9.37
3 2 1	87.80	1.1109	23.62
4 0 0	95.68	1.0392	4.96
3 2 2	99.66	1.0082	12.44
4 1 0	99.66	1.0082	9.70
3 3 0	103.68	0.9798	7.15
4 1 1	103.68	0.9798	8.13
3 3 1	107.77	0.9536	7.48
4 2 0	111.95	0.9295	12.71
4 2 1	116.26	0.9071	23.05
3 3 2	120.74	0.8862	10.33
4 2 2	130.44	0.8485	8.62
4 3 0	135.83	0.8313	9.33
5 0 0	135.83	0.8313	1.36

Table S3. Results of the Rietveld refinement of $Ca(OH)_2$. Cell parameters (Å), cell volume (Å^3) and refined atomic positions are given together with the agreement parameters Rp and Rwp. The peaks position listing the (*hkl*) Miller indices, the 2θ(°) and *d* positions (Å), and relative intensities normalized to 100 (I) are also given.

Unit Cell Parameters			Structure Refinement				Diffraction Data			
a	*c*	*V*	z_O	z_H	Rp	Rwp	*h k l*	*2θ*	*d*	I
3.5947(1)	4.9192(1)	55.048(1)	0.2298(5)	0.419(2)	0.090	0.142	0 0 1	18.02	4.9192	66.60
							1 0 0	28.66	3.1131	17.11
							1 0 -1	34.06	2.6306	100.00
							1 0 1	34.06	2.6306	2.90
							0 0 2	36.51	2.4596	1.61
							1 0 2	47.05	1.9299	20.06
							1 0 -2	47.05	1.9299	27.63
							1 1 0	50.76	1.7973	28.25
							1 1 1	54.30	1.7973	13.72
							0 0 3	56.05	1.6397	1.74
							2 0 0	59.33	1.5565	2.15
							2 0 -1	62.55	1.4840	0.72
							2 0 1	62.55	1.4840	11.05
							1 0 3	64.15	1.4508	9.98
							1 0 -3	64.15	1.4508	1.62
							1 1 2	64.13	1.4512	1.24
							2 0 -2	71.71	1.3153	3.68

2	0	2	71.71	1.3153	5.05
0	0	4	77.57	1.2298	1.92
1	1	3	78.98	1.2114	2.24
2	1	0	81.80	1.1766	2.00
1	0	-4	84.68	1.1438	0.40
1	0	4	84.68	1.1438	1.83
2	0	-3	86.06	1.1289	3.40
2	0	3	86.06	1.1289	0.64
2	1	1	84.63	1.1444	0.34
2	1	-1	84.63	1.1444	3.56
2	1	-2	93.07	1.0614	4.69
2	1	2	93.07	1.0614	3.43
1	1	4	98.75	1.0150	4.73
3	0	0	95.87	1.0377	3.16
3	0	-1	98.70	1.0153	1.17
3	0	1	98.70	1.0153	1.17
0	0	5	103.07	0.9838	0.68
2	0	4	105.94	0.9650	0.26
2	0	-4	105.94	0.9650	1.00
2	1	-3	107.38	0.9560	0.45
2	1	3	107.38	0.9560	2.19
3	0	2	107.36	0.9561	0.23
3	0	-2	107.36	0.9561	0.23
1	0	5	110.40	0.9381	0.22
1	0	-5	110.40	0.9381	1.67
2	2	0	118.01	0.8987	2.42

Table S4. Results from XRPD data refinement by Rietveld method [**Error! Reference source not ound.**]. Together with the wt % for each mineral phase, the accordance parameters Rwp and Rp are reported (%). Polymorphs of the same phase were summed up giving the total abundance of the mineral.

Sample	C3S	C2S	C4AF	C3A	CaO+Ca(OH)2	Gehlenite	Amorphous	Rwp	Rp
**PC1450c**	56.7(7)	8.5(2)	2.57 (6)	12.07(1)	1.57(4)	0.32(3)	18.3 (5)	6.05	4.65
**nPC1450d**	48.7(5)	10.8(2)	0.82(1)	11.11(1)	0.64 (3)	0.54(3)	27.4(4)	5.92	4.50
**PC1350c**	51.7(7)	15.4(2)	2.45 (7)	13.80(1)	3.74(5)	0.27(4)	12.7 (5)	5.20	4.02
**nPC1350dA**	48.9(8)	21.7(3)	0.36(4)	13.66(1)	2.21(5)	0.48(3)	12.7(6)	5.72	4.37
**nPC1350dB**	44.3(7)	16.1(2)	0.91(5)	12.58(1)	2.55(5)	0.32(2)	23.2(5)	6.58	5.06

**nPC1350dC**	43.7(6)	18.2(2)	1.69(5)	11.28(1)	1.93(4)	0.15(2)	23.1(4)	6.18	4.81
**PC1250c**	16.5(3)	42.8(3)	5.35(7)	8.85(6)	12.39(1)	1.40(4)	12.7(4)	6.46	4.90
**PC1250c_2**	17.0(2)	34.8(2)	4.46 (5)	8.32(5)	10.21(8)	1.34(4)	23.8(2)	6.06	4.63
**nPC1250d**	14.0(2)	40.9(2)	5.26(5)	7.53(4)	9.98(7)	2.11(4)	20.2(2)	5.42	4.16
**nPC1250d_2**	12.9(2)	42.6(2)	5.16(5)	7.10(4)	11.19(7)	1.78(4)	19.2(2)	5.68	4.31

© 2018 by the author. Licensee MDPI, Basel, Switzerland. This article is an open access article distributed under the terms and conditions of the Creative Commons Attribution (CC BY) license (http://creativecommons.org/licenses/by/4.0/).
